# Supplementary material for: Neural mechanisms of feature binding in working memory
Source: Commun Biol. 2026 Jan 24;9:270. doi: 10.1038/s42003-026-09548-4 (PMC12916948; doi:10.1038/s42003-026-09548-4)
Supplement: Supplementary file 2 — Supplementary information [file 42003_2026_9548_MOESM2_ESM.pdf]

Supplementary information for "Neural mechanisms of feature bindings in working memory" by Yang Cao, Fuyong Chen, Hao Wang, Xuchu Weng, Jan Theeuwes, & Benchi Wang

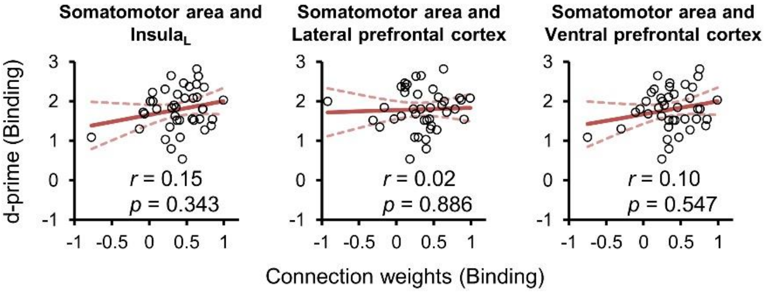

Figure S1. Scatter plots indicate the correlation between connection weights and mean d-prime for specific connections, between somatomotor area and left insula, somatomotor area and lateral prefrontal cortex, somatomotor area and ventral prefrontal cortex in the binding condition. Solid lines indicate linear fits to the data of n = 40 participants, with the shaded areas depicting the 95% confidence interval of the fitted lines.

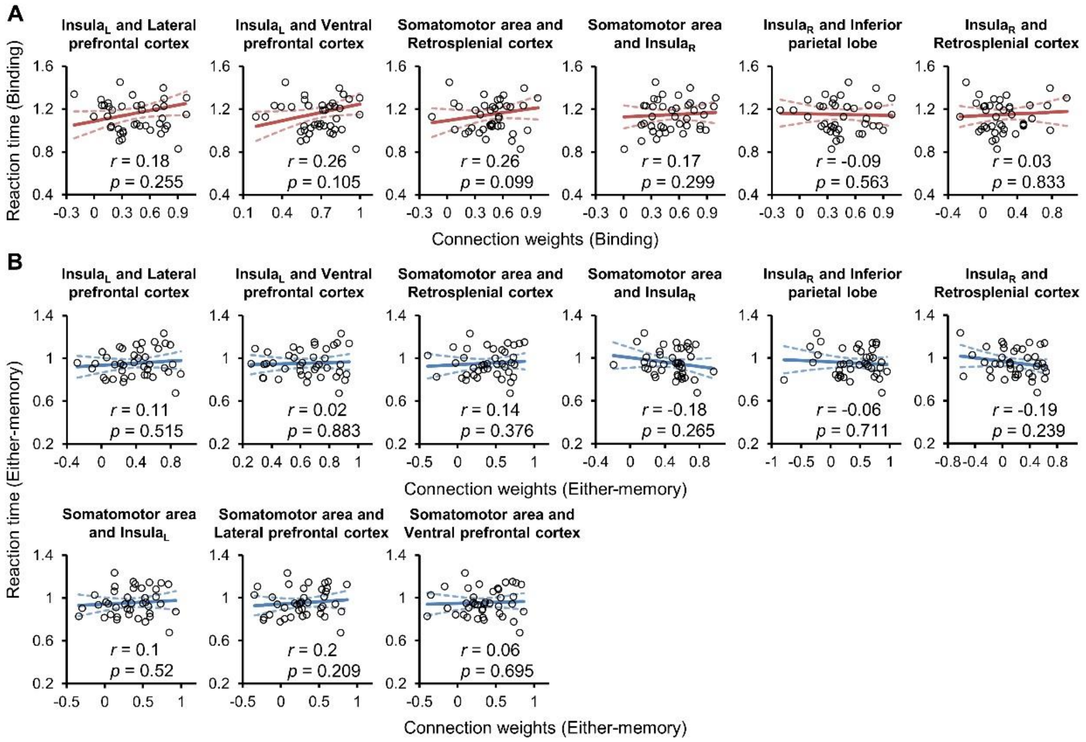

Figure S2. Correlation between mean reaction times (RTs) and connection weights under the binding **(A)** and either-memory **(B)** conditions. Solid lines indicate linear fits to the data of n = 40 participants, with the dotted lines depicting the 95% confidence interval of the fitted lines.

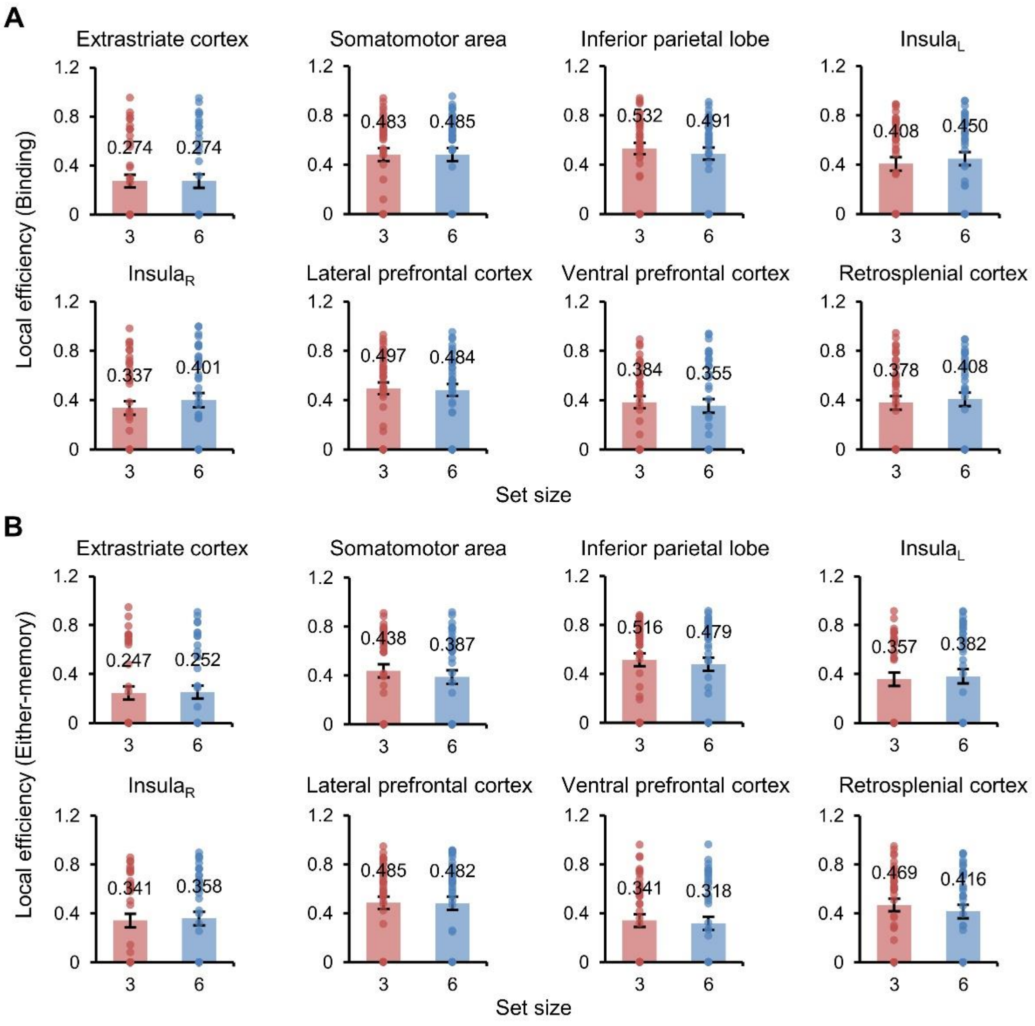

Figure S3. The local efficiency of eight core brain regions was compared between different set sizes under the binding condition (A) and either-memory (B) condition (n = 40 participants). Each solid dot within the bar plots represents a participant and the data variance is represented by ±1 s.e.m.

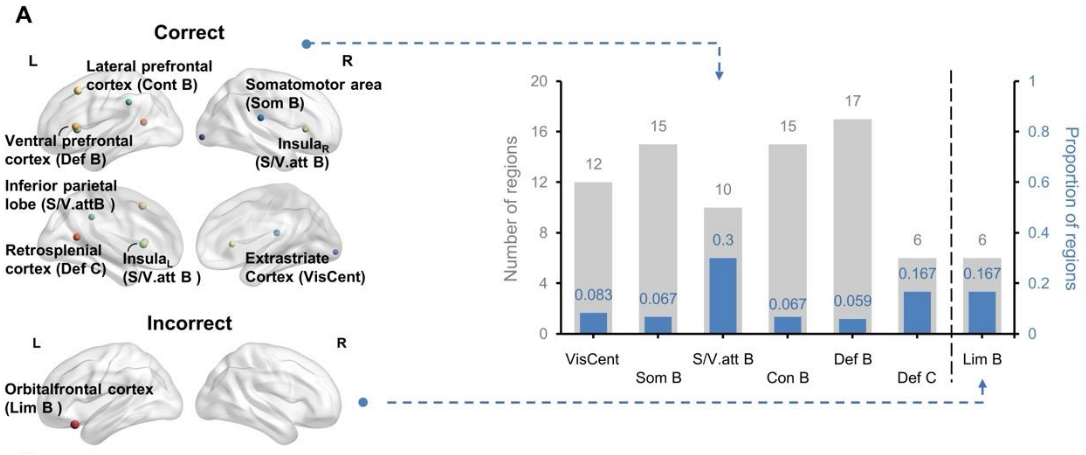

VisCent: Visual central network, Som B: Somatomotor B network; S/V.att B: Salience/Ventral Attention B network; Con B: Control B network; Def B: Default B network; Def C: Default C network; Lim B: Limbic B network.

Figure S4. Left panel shows brain regions exhibiting significantly increased local efficiency in the performance matched subsample for the binding condition compared to the either-memory condition (n = 17 participants), $p < .05$, FDR correction. Right panel indicates the proportion of these brain regions within their corresponding brain network. The gray columns represent the number of brain regions contained in each network, and the blue columns represent the corresponding proportion of brain regions within each network (e.g., 0.3 indicates that three out of ten regions were detected with increased local efficiency in the Salience/Ventral Attention B network for feature binding).

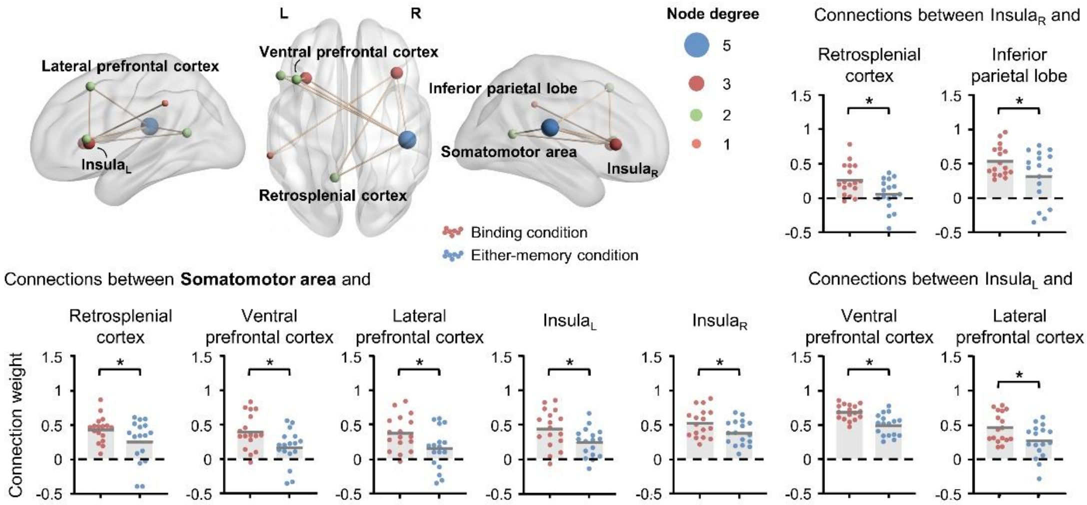

Figure S5. The surface plot shows nine edges connecting seven brain regions that exhibit differences between performance matched subsample for the binding and either-memory condition (n = 17 participants). Node degree is defined as the number of edges directly connected to a specific region. The bar plots show the difference in connection weights for each edge between performance matched subsample for the binding and either-memory condition, *$p < .05$, FDR correction. The horizontal bars indicate mean values and each dot represent a participant.

Validation of the present results

To examine the stability of our results across different brain templates, the Schaefer 400 parcel parcellation was also used to define nodes (ROIs) for constructing functional connectivity (FC) network. We detected a significant increase in local efficiency for the binding condition compared to the either-memory condition ($p < .05$, FDR correction; Fig. S6A) across six brain regions for correct trials, including somatomotor area_1 and somatomotor area_2 (Somatomotor B network), insula_1 and insula_2 (Salience/Ventral Attention A and B network), ventral lateral prefrontal cortex (Salience/Ventral Attention B network) and ventral prefrontal cortex (Default B network). And two other regions were detected for incorrect trials, consisting of medial prefrontal cortex (Default A network) and temporal-parietal area (Temporal parietal network). These findings again indicate that information transfer across a broader brain network, centered around somatomotor area, insula and prefrontal cortex, making important contributions to feature binding.

Subsequently, we extracted a subnetwork from the 400×400 FC matrix for each participant, focusing on six core brain regions (somatomotor area_1, somatomotor area_2, insula_1, insula_2, ventral lateral and ventral prefrontal cortex) that exhibited significant differences in local efficiency between conditions, as identified in correct trials. Network-based statistic (NBS) discovered a significant connected component involving five brain regions (somatomotor area_1, somatomotor area_2, insula_1, insula_2, ventral prefrontal cortex) and six edges, favoring feature binding ($p < .05$, permutation test; Fig. S6B). Among these regions, the majority of significant edges originated from the somatomotor area (5). These findings highlight the importance of somatomotor area and suggest that regional cooperation achieved by interactions between somatomotor area, insula, and prefrontal cortex is crucial during feature binding.

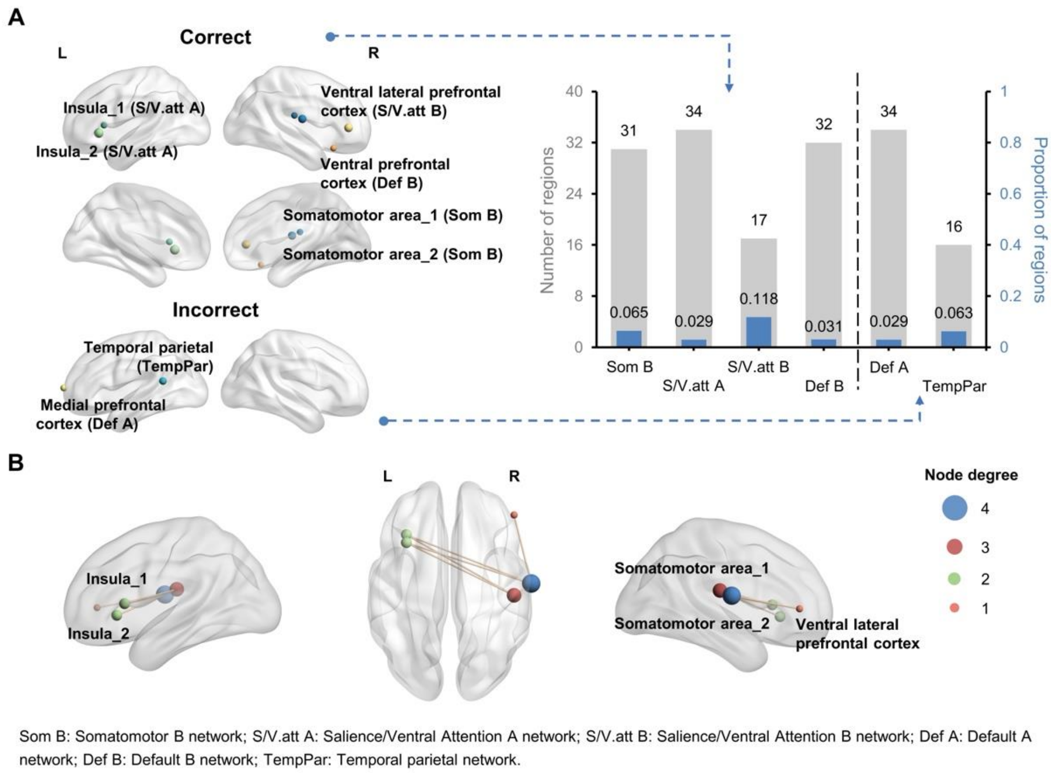

Figure S6. (A) Left panel shows brain regions exhibiting significantly increased local efficiency in the binding condition compared to the either-memory condition (n = 40 participants), $p < .05$, FDR correction. Right panel indicates the proportion of these brain regions within their corresponding brain network. The gray columns represent the number of brain regions contained in each network, and the blue columns represent the corresponding proportion of brain regions within each network. **(B)** The surface plot shows six edges connecting five brain regions that exhibit differences between the binding and either-memory conditions (n = 40 participants). Node degree is defined as the number of edges directly connected to a specific region.

We further estimated the autocorrelation function (ACF) values across six brain regions and found that the activity of somatomotor area exhibited the most rapid decay of ACF with time (a larger slope before ACF hit zero), whereas other regions were correlated across slightly longer periods (Fig. S7A). Then, the intrinsic timescale (sum of ACF values within the specific period) was calculated for each brain region during feature binding. The results showed that somatomotor area had a significant shorter timescale than other regions (all ps < .05, FDR correction; Fig. S7B); while other regions showed a gradual increase in timescale. This hierarchy structure of intrinsic timescale suggests that somatomotor area took part in feature binding in a more dynamic fashion and showed rapid responses to binding signals throughout the task, while other regions processed binding information comparably steadily albeit later in time.

When correlating observed connection weights with mean RTs, we observed significantly positive (Spearman) correlations for connections between somatomotor area_1 and insula_1, $r = 0.45$, $p = .004$, between somatomotor area_1 and insula_2, $r = 0.57$, $p < .001$, between somatomotor area_2 and insula_2, $r = 0.4$, $p = .011$, and between somatomotor area_2 and ventral lateral prefrontal cortex, $r = 0.59$, $p < .001$ (see Fig. S7C). These correlations indicates that extra binding processes required stronger functional connections and also highlight the more important role of somatomotor area in feature binding.

Overall, the results were similar to the Schaefer 200 parcel parcellation, confirming the stability of the observations in the present study.

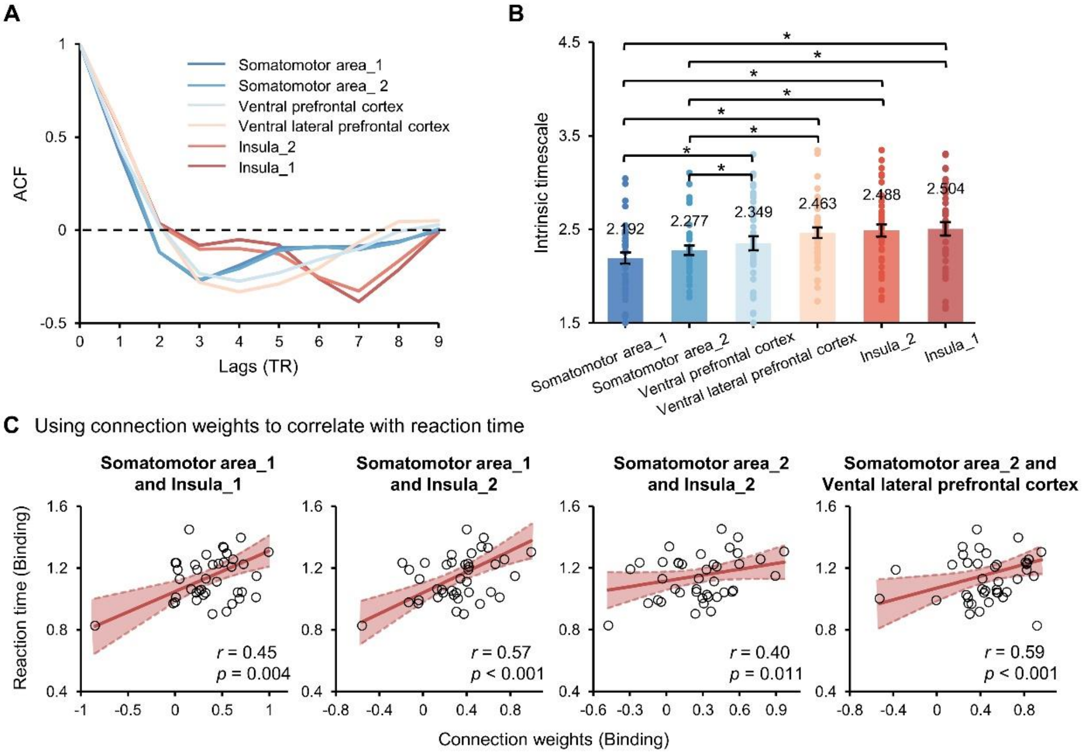

Figure S7. (A) The autocorrelation function (ACF) of six core brain regions estimated during feature binding. **(B)** The bar plot displays intrinsic timescale of six regions in the binding condition ($n = 40$ participants). The somatomotor area had significantly shorter timescale than the other regions, * $p < .05$, ** $p < .01$, *** $p < .001$, FDR correction. Each solid dot within the bar plots represents a participant and the data variance is represented by ±1 s.e.m. **(C)** Scatter plots indicate the correlation between connection weights and mean RTs for specific connections, between somatomotor area_1 and insula_1, somatomotor area_1 and insula_2, somatomotor area_2 and insula_2, somatomotor area_2 and ventral lateral prefrontal cortex in the binding condition. Solid lines indicate linear fits to the data of $n = 40$ participants, with the shaded areas depicting the 95% confidence interval of the fitted lines.

Additionally, to examine the stability of our results across different connectivity

computing approach, we applied a fixed-density thresholding approach (at 10% sparsity) to construct connectivity networks and calculate local efficiency (E_{loc}) per participant with the Schaefer 200 parcel parcellation. This analysis revealed a significant increase in efficiency in the binding condition versus the either-memory condition ($p < .05$, FDR corrected). Notably, this effect was observed in six brain regions—including the extrastriate cortex (Visual central network), somatomotor area (Somatomotor B network), insula (Salience/Ventral Attention B network), ventral prefrontal cortex (Default B network), dorsal prefrontal cortex (Default A network), and temporal pole (Limbic A network)—which are consistent with observed feature binding regions in our study. The convergence of results across methods further supports the robustness of our findings.

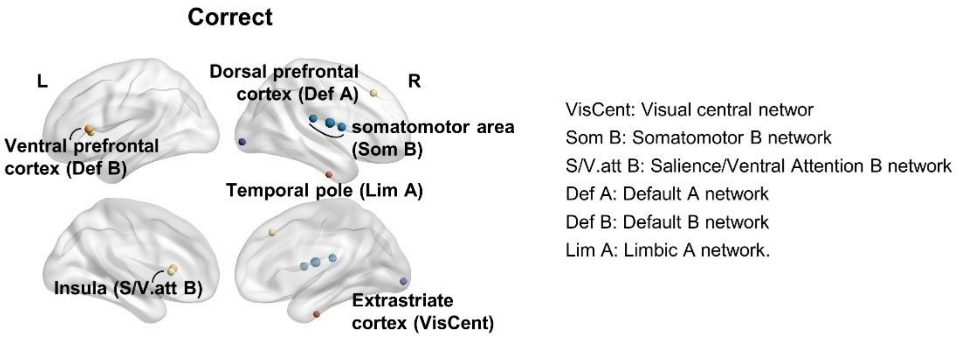

Figure S8. Brain regions exhibiting significantly increased local efficiency in the binding condition compared to the either-memory condition using the fixed-density thresholding approach (with 10 % sparsity, $n = 40$ participants), $p < .05$, FDR correction.
