# Supplementary material for: Neural mechanisms of feature binding in working memory
Source: Commun Biol. 2026 Jan 24;9:270. doi: 10.1038/s42003-026-09548-4 (PMC12916948; doi:10.1038/s42003-026-09548-4)
Supplement: Supplementary file 3 — Reporting-summary [file 42003_2026_9548_MOESM3_ESM.pdf]

nature portfolio

Corresponding author(s): Benchi Wang, Hao Wang, Xuchu Weng

Last updated by author(s): Dec 14, 2025

Reporting Summary

Nature Portfolio wishes to improve the reproducibility of the work that we publish. This form provides structure for consistency and transparency in reporting. For further information on Nature Portfolio policies, see our Editorial Policies and the Editorial Policy Checklist.

Statistics

For all statistical analyses, confirm that the following items are present in the figure legend, table legend, main text, or Methods section.

n/a	Confirmed	
☐	☒	The exact sample size (*n*) for each experimental group/condition, given as a discrete number and unit of measurement
☐	☒	A statement on whether measurements were taken from distinct samples or whether the same sample was measured repeatedly
☐	☒	The statistical test(s) used AND whether they are one- or two-sided<br>*Only common tests should be described solely by name; describe more complex techniques in the Methods section.*
☐	☒	A description of all covariates tested
☐	☒	A description of any assumptions or corrections, such as tests of normality and adjustment for multiple comparisons
☐	☒	A full description of the statistical parameters including central tendency (e.g. means) or other basic estimates (e.g. regression coefficient) AND variation (e.g. standard deviation) or associated estimates of uncertainty (e.g. confidence intervals)
☐	☒	For null hypothesis testing, the test statistic (e.g. *F*, *t*, *r*) with confidence intervals, effect sizes, degrees of freedom and *P* value noted<br>*Give P values as exact values whenever suitable.*
☒	☐	For Bayesian analysis, information on the choice of priors and Markov chain Monte Carlo settings
☒	☐	For hierarchical and complex designs, identification of the appropriate level for tests and full reporting of outcomes
☐	☒	Estimates of effect sizes (e.g. Cohen's *d*, Pearson's *r*), indicating how they were calculated

Our web collection on statistics for biologists contains articles on many of the points above.

Software and code

Policy information about availability of computer code

Data collection	Data were collected using a 3T Siemens Trio scanner with a 20-channel phase-array coil. The graphical user interface (GUI) of the experimental task was developed in Python 3.8.
Data analysis	Statistical analysis were performed using MATLAB R2018b, SPM12 (version 7771), the Brain Connectivity Toolbox and BrainNet Viewer.

For manuscripts utilizing custom algorithms or software that are central to the research but not yet described in published literature, software must be made available to editors and reviewers. We strongly encourage code deposition in a community repository (e.g. GitHub). See the Nature Portfolio guidelines for submitting code & software for further information.

Data

Policy information about availability of data

All manuscripts must include a data availability statement. This statement should provide the following information, where applicable:

- Accession codes, unique identifiers, or web links for publicly available datasets
- A description of any restrictions on data availability
- For clinical datasets or third party data, please ensure that the statement adheres to our policy

The processing codes and derived fMRI data will be made available upon reasonable request. Any data provided must be kept confidential and must not be shared with others without approval.

Research involving human participants, their data, or biological material

Policy information about studies with human participants or human data. See also policy information about sex, gender (identity/presentation), and sexual orientation and race, ethnicity and racism.

Reporting on sex and gender	Sex and gender were not considered in the present study, as we assumed that they all have the same cognitive processing.
Reporting on race, ethnicity, or other socially relevant groupings	Not applicable.
Population characteristics	We have forty volunteers (18 males, aged 19.45±1.21 years).
Recruitment	Participants were recruited from South China Normal University. Enrollment was entirely dependent on the voluntary principle. Thus, we do not expect any significant self-selection bias in this study.
Ethics oversight	The Institutional Review Board of South China Normal University (2020-3-013).

Note that full information on the approval of the study protocol must also be provided in the manuscript.

Field-specific reporting

Please select the one below that is the best fit for your research. If you are not sure, read the appropriate sections before making your selection.

☒ Life sciences ☐ Behavioural & social sciences ☐ Ecological, evolutionary & environmental sciences

For a reference copy of the document with all sections, see nature.com/documents/nr-reporting-summary-flat.pdf

Life sciences study design

All studies must disclose on these points even when the disclosure is negative.

Sample size	Sample size was predetermined to be approximately twice that of comparable neuroscience studies investigating feature binding (with sample sizes ranged from 5 to 22; see Libby et al., 2014; Parra et al., 2014; Seymour et al., 2010) and forty participants would be enough to replicate the previous behavioral findings (Wheeler & Treisman, 2002) with a power larger than 0.99.
Data exclusions	No data exclusion.
Replication	We indeed used multiple methods with slightly changed parameters to re-analyze the data, all results were consistent.
Randomization	All participants needed finish all the testing conditions, it is a within-subjects design.
Blinding	We did not have group allocation.

Reporting for specific materials, systems and methods

We require information from authors about some types of materials, experimental systems and methods used in many studies. Here, indicate whether each material, system or method listed is relevant to your study. If you are not sure if a list item applies to your research, read the appropriate section before selecting a response.

Materials & experimental systems

n/a	Involved in the study	
☒	☐	Antibodies
☒	☐	Eukaryotic cell lines
☒	☐	Palaeontology and archaeology
☒	☐	Animals and other organisms
☒	☐	Clinical data
☒	☐	Dual use research of concern
☒	☐	Plants

Methods

n/a	Involved in the study	
☒	☐	ChIP-seq
☒	☐	Flow cytometry
☐	☒	MRI-based neuroimaging

Plants

Seed stocks	Not applicable
Novel plant genotypes	Not applicable
Authentication	Not applicable

Magnetic resonance imaging

Experimental design

Design type	event-related design
Design specifications	The experiment comprised two conditions. For each condition, participants completed a practice run consisting of 16 trials before fMRI scanning, and three runs of 24 trials during fMRI scanning.
Behavioral performance measures	Correct button press and reaction time were recorded. According to these two measures, we could confirm whether the participants were performing the task as expected.

Acquisition

Imaging type(s)	functional and structural
Field strength	3T
Sequence & imaging parameters	For the acquisition of echo-planar imaging (EPI) images, a gradient EPI sequence with a TE (echo time) of 30 ms and a TR (repetition time) of 1500 ms was employed. The EPI sequence comprised 46 axial slices with a 3 mm slice thickness, a 64×64 acquisition matrix, and a field of view (FOV) of 192×192 mm. High-resolution anatomical images were also acquired for each participant using a T1-weighted MPRAGE sequence with a TE of 2.27 ms, a TR of 1900 ms, a flip angle of 7°, and an FOV of 256×256 mm. The MPRAGE sequence featured an isometric voxel resolution of 1 mm and covered the whole brain with 208 sagittal slices.
Area of acquisition	whole brain scan.

Diffusion MRI ☐ Used ☒ Not used

Preprocessing

Preprocessing software	SPM12, version 7771 (https://www.fil.ion.ucl.ac.uk/spm/software/spm12/), was used for the analysis of the imaging data. All EPI images were slice-time-corrected, motion-corrected, registered to the Montreal Neurological Institute (MNI) template, spatially smoothed (with an isotropic 6 mm full-width at half-maximum Gaussian filter), and high-pass filtered (128 s).
Normalization	Liner normalization.
Normalization template	Montreal Neurological Institute (MNI) template.
Noise and artifact removal	Participants exhibiting high levels of motion, defined by a mean Frame-wise displacement (FD) greater than 0.5 mm, were excluded. No participants were excluded due to excessive movement during the MRI scans.
Volume censoring	Not applicable.

Statistical modeling & inference

Model type and settings	A first level general linear model (GLM) was applied individually on the preprocessed functional data. Event-related regressors were obtained by convolving the onset of each trial with the canonical hemodynamic response function (HRF), capturing the expected BOLD response to each trial. And six head-movement-related regressors were also included in the model to account for motion artifacts. Subsequently, the second-level GLM was performed to complete a group analysis, calculating brain activations.
Effect(s) tested	ANOVA was used to define precise effect of stimulus conditions.

Specify type of analysis: ☐ Whole brain ☐ ROI-based ☒ Both

Anatomical location(s)	The Schaefer 200 parcels with 17 networks (Schaefer et al., 2018; MNI-3mm) was adopted to define the regions of interest (ROIs).
Statistic type for inference<br>(See Eklund et al. 2016)	In the whole brain analysis, the threshold of statistical maps was set to p < .001 and corrected them using cluster family-wise error (FWE) correction. In the ROI-based analysis, t-tests and False Discovery Rate (FDR) correction were used to detect differences.
Correction	FWE, FDR and permutation.

Models & analysis

n/a	Involved in the study
☐	☒ Functional and/or effective connectivity
☐	☒ Graph analysis
☒	☐ Multivariate modeling or predictive analysis

Functional and/or effective connectivity	Pearson correlations.
Graph analysis	The weighted connectivity network was used to compute the efficiency-based properties (i.e., local efficiency) and applied network-based statistic (NBS) to detect subnetworks. All analysis were performed in the subject level.
